# Validity and Reliability Analysis of the Artificial Intelligence–Digital Life Balance Scale

**DOI:** 10.1007/s11126-025-10167-1

**Published:** 2025-06-04

**Authors:** Nuri Erdemir, Servet Atik

**Affiliations:** 1 Department of Counselling & Guidance, Faculty of Education, İnönü University, Malatya, TR 44800 Türkei; 2 Department of Educational Administration, Faculty of Education, İnönü University, Malatya, TR 44800 Türkei

**Keywords:** Digital life balance, Artificial intelligence, Psychometric scale, Mental health, Digital well-being, University students

## Abstract

This study aimed to develop and validate the Artificial Intelligence - Digital Life Balance Scale (AI-DLBS), a psychometric tool designed to assess the multidimensional impact of digital technologies and artificial intelligence (AI) on individuals’ psychological, social, physical, and academic well-being. Utilizing ChatGPT-4, a novel AI-driven approach, the 40-item scale was constructed to measure five key dimensions: frequency and duration of digital device use, psychological and social effects, physical health impacts, academic performance, and technology access and dependency. Data were collected from three independent samples of university students in Turkey (*N* = 773, *N* = 325, *N* = 86) using convenience sampling. Exploratory and confirmatory factor analyses revealed a six-factor structure, explaining 60.83% of the variance, with acceptable model fit indices (e.g., RMSEA = 0.06, CFI = 0.90). The scale demonstrated strong internal consistency (Cronbach’s α = 0.68-0.87) and test-retest reliability. The AI-DLBS offers significant potential for psychiatric research and clinical practice, enabling mental health professionals to evaluate technology-related risks, such as anxiety, social isolation, and dependency, and design targeted interventions, including digital detox programs. The innovative use of AI in scale development highlights both its efficiency and ethical challenges, such as data bias risks. Findings suggest the AI-DLBS is a reliable and valid tool for assessing digital life balance, with implications for global mental health research and policy-making. Future studies should validate the scale across diverse populations and cultural contexts.

## Introduction

In the contemporary era, artificial intelligence and digital technologies, which have become deeply embedded in every facet of life, have profoundly transformed individuals’ daily routines, impacting various aspects in numerous ways and highlighting the need for a comprehensive understanding of their effects on individuals’ well-being [1]. These impacts are extensive and complex. In daily life, numerous domains such as communication, access to information, work, and health management have undergone fundamental changes with the widespread adoption of artificial intelligence tools [2–4]. Digital transformation is a global phenomenon, and the use of digital technologies varies according to cultural contexts. For instance, while the social media usage rate among university students in Türkiye exceeds 90% [5], this rate may be lower in European countries, underscoring the universal yet culturally differentiated nature of issues like digital addiction or digital life balance. As artificial intelligence becomes increasingly integrated into technical systems, understanding user responses and perspectives toward AI technologies has become critically important [6]. Measuring and balancing individuals’ reliance on AI recommendations is essential [7]. This digital transformation has introduced new challenges, such as potential negative effects on psychological, social, and physical well-being, while also offering unique opportunities [8]. Excessive use of digital technologies has been associated with various psychiatric issues, including anxiety, depression, attention-deficit/hyperactivity disorder (ADHD), and sleep disorders [9, 10]. In this context, a scale measuring digital life balance provides mental health professionals with a valuable tool to assess individuals’ technology use and develop appropriate interventions. For example, studies conducted in the United States have revealed a positive correlation between screen time and depressive symptoms among young adults [11], while research in Asian countries has reported that social media addiction negatively impacts academic achievement [12]. These findings suggest that a digital life balance scale could contribute significantly to the global mental health literature. The growing influence of digital technologies, fueled by advancements in artificial intelligence, continues to bring both advantages and new challenges to individuals [13]. Rapidly evolving AI technologies have created new perspectives in education, healthcare, and social interactions, increasing the need to understand and mitigate their potential negative impacts on digital well-being [14].

The concept of digital life balance has emerged as a foundational framework for understanding the multifaceted impacts of digital technologies on individuals’ lives. This concept encompasses the equilibrium between technology use and various life domains, such as work, leisure, social interactions, and health [15]. In particular, excessive use of digital devices has been associated with psychiatric risk factors, including social isolation, loneliness, and stress, necessitating the development of balanced technology use habits to safeguard individuals’ mental health [16]. As interactions with digital devices and online platforms increase, maintaining a healthy balance becomes imperative to prevent adverse outcomes such as addiction, stress, social isolation, and physical health issues. Successfully staying healthy while using technology has become a critical skill in today’s world. Digital well-being, in this context, represents an intervention system aimed at preserving this health [17]. Digital well-being encompasses the ability to control technology use and ensure its alignment with an individual’s goals, values, and relationships [18]. The capacity to balance technology use with personal well-being is increasingly vital for adapting to the challenges of the modern world [19]. As efforts to balance the advantages offered by technology with its potential impacts on individuals’ health and happiness intensify, the need to assess digital life balance grows [20]. Despite the recognized importance of digital life balance, reliable and scientifically grounded tools for accurately measuring this balance remain insufficiently developed. Existing tools often focus on specific aspects of digital technology use, such as internet addiction, social media engagement, or smartphone addiction, while neglecting the broader range of factors contributing to an individual’s overall digital balance [21]. For instance, scales developed in Western countries (e.g., Smartphone Addiction Scale; [22] typically focus on digital addiction, whereas this study aims to provide a more comprehensive contribution to the global literature by addressing the multidimensional nature of digital life balance.

The current lack of comprehensive measurement tools capable of addressing individuals’ ability to balance technology use with other aspects of their lives in a multidimensional manner highlights a significant need. In particular, there is a demand for tools that can assess the impact of technology on individuals’ subjective well-being [23]. Contemporary measurements often focus solely on specific issues such as internet addiction, smartphone addiction, or social media use. However, these measurements overlook the broader elements that influence an individual’s digital life [18]. An increasing number of people believe that technology should align with human values and consider individual and societal needs [24]. In this context, a scale evaluating digital life balance could serve as a critical tool for understanding the mental health implications of technology use and developing psychiatric interventions. One of the primary challenges limiting our understanding of the complex relationship between digital technology use and individual well-being is the absence of a holistic evaluation approach. This gap hinders the development of targeted interventions and strategies to support healthier digital lifestyles. To address this gap, there is a need for assessment tools that encompass the multidimensional nature of digital life balance, including the frequency and duration of digital device use, psychological and social impacts, effects on physical health, influence on academic achievement, access to technology, and addiction. Within this framework, the present study aims to develop a new psychometric scale, the Artificial Intelligence - Digital Life Balance Scale, designed to evaluate individuals’ digital life balance in the context of artificial intelligence and digital technology use in a multidimensional manner, thereby addressing a significant gap in this field [25].

This research aims to develop a valid and reliable scale that reflects the multidimensional and complex nature of digital life balance within the context of digital technologies. The developed scale offers a significant and functional assessment tool for researchers, mental health professionals, practitioners, and individuals seeking to understand and enhance their digital well-being. The development of the Artificial Intelligence - Digital Life Balance Scale is driven by the growing need for a reliable and valid instrument to evaluate the impact of digital technologies on various aspects of individuals’ lives. The rapid integration of artificial intelligence into daily tasks and processes, from education to healthcare, necessitates a detailed understanding of how individuals perceive and manage their interactions with these technologies. Furthermore, the scale’s multidimensional structure enables a nuanced evaluation of specific areas where individuals struggle to maintain a healthy digital balance, thereby informing the development of targeted interventions and strategies to promote digital well-being. This study addresses this gap by introducing the Artificial Intelligence - Digital Life Balance Scale, a new 40-item tool designed to assess digital life balance in the context of digital technology use. The Artificial Intelligence - Digital Life Balance Scale comprises five distinct subscales: Frequency and Duration of Digital Device Use, which reflects the extent of individuals’ engagement with digital technologies; Psychological and Social Impacts, which evaluates the effects of digital technology use on mental and emotional well-being and social interactions; Physical Health Impacts, which assesses the physical consequences of prolonged digital device use, such as eye strain, musculoskeletal issues, and sleep disturbances; Impact on Academic Performance, which examines the effects of digital technology use on students’ learning outcomes and academic engagement [26]; and Access to Technology and Addiction, which measures individuals’ dependence on digital technologies and their perceived ability to function without them. Each subscale contains eight items, with two reverse-coded items included in each subscale to reduce response bias and enhance the scale’s psychometric properties. The overarching goal of this study is to provide a detailed account of the development and psychometric evaluation of the Artificial Intelligence - Digital Life Balance Scale, offering researchers and practitioners a reliable and valid tool for assessing digital life balance in the context of digital technology use. Higher scores on the scale indicate a more balanced and positive integration of digital technologies into an individual’s life, reflecting a harmonious relationship between the digital and offline worlds. Specifically, this research aims to rigorously examine the scale’s psychometric properties, including construct validity, internal consistency reliability, and test-retest reliability.

## Materials and Methods

### Working Group

The validity and reliability analyses of the scale were conducted using three distinct sample groups. These groups were formed through convenience sampling, a method known for its simplicity, cost-effectiveness, and speed [27, 28].

*The first study group* was used to collect data for Exploratory Factor Analysis (EFA) to examine the construct validity of the scale. This group consisted of 773 students enrolled at a university in eastern Türkiye during the 2024–2025 academic year. It is noted that a sample size of 500 or more is considered highly suitable for EFA [29, 30]. Of the students in this group, 538 were female (69.6%) and 235 were male (30.4%). The distribution of students by academic year was as follows: 130 (16.8%) first-year, 214 (27.7%) second-year, 246 (31.83%) third-year, and 183 (23.67%) fourth-year and above.

*The second study group* was used to collect data for Confirmatory Factor Analysis (CFA) and to calculate criterion-related validity. This group comprised 325 students enrolled in a faculty of education in eastern Türkiye during the 2024–2025 academic year. In the literature, a sample size of 200 to 400 is generally considered appropriate for CFA [31]. Of the students in this group, 224 were female (68.9%) and 101 were male (31.1%).

*The third study group* was used to conduct the test-retest reliability analysis. This group consisted of 86 students enrolled in a faculty of education in eastern Türkiye during the 2024–2025 academic year. Of the students in this group, 56 were female (65.1%) and 30 were male (34.9%).

### Data Collection Tools

#### Artificial Intelligence - Digital Life Balance Scale

The primary objective of this research is to develop a valid and reliable measurement tool that holistically evaluates the psychological, social, physical, and academic dimensions of digital life balance. To this end, the paid version of the language model ChatGPT-4, developed by OpenAI, was utilized to create the Digital Life Balance Scale (DYDÖ). The scale was directly generated using Turkish prompts through artificial intelligence, aiming to provide a practical example that practitioners with limited resources (e.g., school counselors, educators) can access and implement.

The prompt provided to ChatGPT-4 was as follows:

*Digital Life Balance Scale (DLBS): This scale can serve as a tool to assess digital addiction and its effects on an individual’s psychological*,* social*,* physical*,* and academic functioning. The subscales of the scale can be designed as follows: Frequency and duration of digital device use*,* Psychological and social impacts*,* Physical health impacts*,* Impact on academic performance/success*,* and Access to and dependence on technology. Based on this information*,* create a 7-point Likert-type scale. Ensure that two items in each subscale are reverse-scored. Write 8 items for each subscale. Higher scores from the subscales and the total scale should indicate a better digital life balance for the individual*,* and the items should be written accordingly.*

This prompt was designed to test the productivity, accessibility, and transparency of artificial intelligence technologies in the scale development process. The prompt was intentionally kept broad and free from extensive theoretical guidance to simulate a scenario in which users with limited theoretical backgrounds can effectively utilize artificial intelligence.

The initial version of the scale was prepared by starting a new session to ensure it was not influenced by prior user data, based on the items generated by ChatGPT-4. Among the methodological advantages provided by ChatGPT-4 in the scale development process, rapid item generation and accessibility stand out, while limitations include a lack of critical thinking and dependence on training data. For instance, items generated by artificial intelligence may lack a theoretical foundation or fail to fully align with cultural contexts [32]. To mitigate such risks, the scale items were meticulously evaluated by three faculty members (experts in Guidance and Psychological Counseling and Turkish Language Education). The experts reviewed each item based on criteria such as theoretical appropriateness, linguistic clarity, cultural relevance, and the balance of reverse-scored items. However, in line with the study’s objectives, no content changes were made to the original items generated by the AI. This expert evaluation process strengthened the theoretical foundations of the scale and confirmed that the items accurately reflected the concept of digital life balance. During the evaluation, criteria such as alignment with the theoretical framework, clarity of expression, conciseness, and appropriateness of reverse scoring were considered [33, 34]. This academic validation process served as a critical filter to reduce the risk of potential content errors or cultural misalignment in the AI-generated items. Nevertheless, consistent with the study’s purpose of examining the validity and reliability analyses of a psychometrically developed AI-based measurement tool, the scale was used in its original form without any modifications.

The Digital Life Balance Scale consists of a total of 40 items organized into 5 subscales. Each subscale contains 8 items, 2 of which are structured to be reverse-scored. The items are designed to measure the positive contributions of digital technologies to an individual’s life, while the reverse-scored items include negative statements indicating digital imbalance or excessive use.

The subscales are as follows:

- Frequency and duration of digital device use (Items 1–8).
- Psychological and social impacts (Items 9–16).
- This subscale aims to assess the effects of digital technology use on an individual’s mental health, focusing particularly on its association with psychiatric outcomes such as anxiety, stress, loneliness, and social withdrawal. For example, the item “I can say that I do not prioritize my relationships in virtual communication networks over my real-life relationships” measures the risk of social isolation, while reverse-scored items (e.g., “I frequently feel stressed due to digital device use”) are designed to evaluate the psychological burden caused by technology [16].
- Physical health impacts (Items 17–24).
- Impact on academic performance/success (Items 25–32).
- Access to and dependence on technology (Items 33–40).

All items are structured on a 7-point Likert scale (1 = Strongly Disagree, 7 = Strongly Agree). Subscale scores reflect the individual’s digital life balance in the respective domain, while the total score represents the individual’s overall digital life balance. A higher score indicates that the individual uses digital technology in a balanced, healthy, and functional manner. Additionally, a similarity check for the scale was conducted via Turnitin, and the report for the document submitted on May 3, 2025, indicated a similarity rate of 0% (Submission ID: 2664690276). This result supports the originality of the scale.

### Analysis of Data

In the study, statistical analysis of the data obtained from the scale was conducted using SPSS for Windows 27.0 and AMOS 21.0 software packages. To ensure the content validity of the scale, expert opinions from professionals in the field were sought. For construct validity, Exploratory Factor Analysis (EFA) was applied to the first sample group, while Confirmatory Factor Analysis (CFA) was conducted on the second sample group. Item-Test Correlation analysis was performed based on the EFA data.

In examining the normality assumption of the datasets for EFA and CFA, the assumptions that skewness and kurtosis values should fall between + 1 and − 1, and Z-scores should range between + 3 and − 3, were adopted [35]. The analyses revealed that the datasets used for both EFA and CFA exhibited normal distribution. The reliability of the scale was tested using data collected for EFA, employing Cronbach’s alpha, McDonald’s omega composite reliability coefficients, composite reliability (CR), and average variance extracted (AVE) coefficients. An α value above 0.7 is considered acceptable.

In scale development and adaptation studies, factor analysis is employed to determine the construct validity of a scale. Factor analysis includes both exploratory factor analysis (EFA) and confirmatory factor analysis (CFA) [36, 37]. While EFA reveals the conceptually grounded item structure and dimensions of the scale, CFA is used to validate the structure of the scale developed with data collected from a different sample and to assess item quality [38]. The test-retest method was utilized to determine the scale’s temporal stability reliability. At this analysis stage, Pearson correlation coefficients and intraclass correlation coefficients (ICC) were calculated [39].

Measurement invariance was investigated using multi-group confirmatory factor analysis (MG-CFA) [40, 41]. Measurement invariance provides information about the psychometric equivalence of a construct across groups or over time [42]. In this study, measurement invariance was tested at the configural, metric, and scalar invariance levels, in that order [43, 44].

### Findings 

#### Exploratory Factor Analysis

As part of the validity studies for the Artificial Intelligence - Digital Life Balance Scale, Exploratory Factor Analysis (EFA) was conducted first. Prior to the EFA, Kaiser-Meyer-Olkin (KMO) and Bartlett’s Test of Sphericity were performed. The results of the KMO test (= 0.931) and Bartlett’s test (= 9097.690, *p* =.000) indicated that the KMO value, being above 0.60, confirmed the suitability of the data for factor analysis [45]. Consequently, it was deemed appropriate to proceed with the Exploratory Factor Analysis. The results of the EFA are presented in Table 1.

**Table 1:** Exploratory factor analysis of the artificial intelligence - digital life balance scale

Items	Dimensions						Factor Common Variance	Corrected Item-Test Correlations
	Factor 1	Factor 2	Factor 3.	Factor 4	Factor 5	Factor 6		
1.I believe I can plan my daily technological device usage time in accordance with my needs.	,**690**						,572	0.808
2.I take care to use my digital devices only when necessary.	,**788**						,684	0.854
3.Even when I turn to digital devices to spend my free time, I do not excessively prolong the duration.	,**736**						,603	0.835
4.Every day I use technological devices, I can clearly define the purpose of my usage.	,**683**						,613	0.783
7.I can say that my digital device usage in my daily planning does not disrupt my other responsibilities.	,**528**						,504	0.830
8.When managing time related to technology, I can clearly establish my personal boundaries.	,**581**						,561	0.797
10.I can maintain my social relationships face-to-face while balancing them effectively with digital communication.		,**628**					,571	0.776
12.I make an effort to frequently spend time with family members and friends outside of technology use.		,**686**					,635	0.800
15.I can say that I do not consider my relationships in virtual communication networks more important than my real-life relationships.		,**735**					,610	0.706
17.I take regular breaks to avoid prolonged inactivity while using technology.			,**553**				,451	0.598
20. I take precautions to reduce physical discomfort caused by spending extended periods in front of a screen.			,**704**				,649	0.716
22.I can say that I pay attention to ergonomic conditions while using technological devices.			,**759**				,692	0.723
23.I take care not to disrupt my regular exercise and healthy eating habits while planning my technology use.			,**670**				,636	0.748
24.I closely monitor the effects of digital use on my physical health and can impose limitations when necessary.			,**586**				,643	0.815
21.Technology use frequently leads me to neglect my eating habits or exercise plans.				,683			,483	0.745
27.The time I spend in digital environments hinders my ability to prepare for classes regularly.				,809			,675	0.742
29. I notice that I often postpone my academic responsibilities due to being immersed in technology.				,814			,686	0.721
35. A disruption in internet access significantly disrupts my day.				,639			,495	0.545
25.I believe I can effectively use digital resources in my academic research.					,698		,640	
26.Technology use positively supports my ability to focus on assignments and projects.					,673		,606	
28.In my academic work, I consciously select and use appropriate online tools and library databases.					,710		,609	
30.While spending time on online educational platforms, I act purposefully in line with my learning goals.					,696		,617	
31. I can say that I enrich my learning process through digital materials.					,761		,656	
32. I position technology as a supportive tool to enhance my academic performance.					,780		,697	
33. Even if I am not constantly online, I do not feel uneasy in situations without internet access.						,**696**	,560	
34. In cases where I cannot access my digital devices, I can comfortably carry out my daily tasks using alternative methods.						,**679**	,623	
36. I have full confidence in my ability to maintain my social life without technological devices.						,698	,615	
38. I possess the ability to meet my needs through other means without being dependent on technology.						,677	,645	
**Eigenvalue**	3.45	2.05	2.87	2.28	3.92	2.46		
**Total Variance Explained (60**,**83)**	12.34	7.31	10.27	8.13	13.99	30.05		
**Cronbach Alpha**	0.90							

**Fig. 1:**
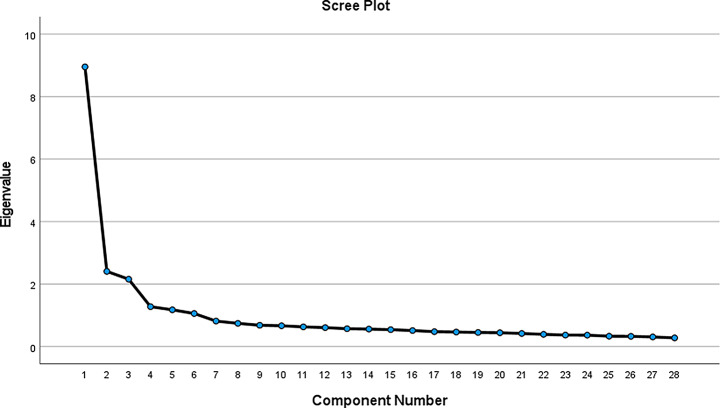
Factor analysis line graph

Upon examining the results of the Exploratory Factor Analysis (EFA), it was determined that the factor loadings of the items ranged between 0.53 and 0.81, while the common factor variances varied between 0.48 and 0.69. The analysis revealed that the total explained variance of the scale was 60.83%, with a six-factor structure having eigenvalues greater than 1. Initially, the scale was structured with five subscales: Frequency and Duration of Digital Device Use, Psychological and Social Impacts, Physical Health Impacts, Impact on Academic Performance, and Access to and Dependence on Technology. However, the EFA results indicated a six-factor structure. This structural differentiation can be attributed to the division of the Access to and Dependence on Technology subscale into two distinct factors. The fifth factor reflects the conscious use of digital resources to support academic performance (e.g., “*I believe I can effectively use digital resources in my academic research*”), while the sixth factor pertains to the level of dependence on technology and offline functionality (e.g., “*In cases where I cannot access my digital devices*,* I can comfortably carry out my daily tasks using alternative methods*”). This divergence highlights the need to address the positive and negative impacts of digital technology on individuals’ lives as separate dimensions [46]. The graph in Fig. 1 also indicates that the scale begins to break after the sixth dimension, confirming the six-factor structure. Based on these findings, it can be concluded that the scale has a six-dimensional structure [47].

Using the EFA data, Cronbach’s alpha (α), McDonald’s Omega reliability coefficients, composite reliability (CR), and average variance extracted (AVE) coefficients were calculated for the scale. Composite Reliability (CR) and AVE were used to test the convergent validity of the Artificial Intelligence - Digital Life Balance Scale. The results are shown in Table 2.

**Table 2:** Reliability analysis of the artificial intelligence - digital life balance scale

Dimensions	Cronbach’s α	McDonald’s Omega	CR	AVE
1	0.84	0.84	0.83	0.51
2	0.68	0.68	0.73	0.50
3	0.83	0.83	0.79	0.51
4	0.73	0.73	0.83	0.58
5	0.87	0.87	0.87	0.59
6	0.79	0.79	0.78	0.50

It was determined that the Cronbach’s alpha coefficients for the dimensions of the Artificial Intelligence - Digital Life Balance Scale ranged between 0.68 and 0.87, and the McDonald’s Omega coefficients ranged between 0.68 and 0.87. Generally, a Cronbach’s alpha internal consistency coefficient and McDonald’s Omega coefficient of 0.70 or higher indicate that the measurement tool is reliable [48, 49]. Based on these findings, it was concluded that the items comprising the Artificial Intelligence - Digital Life Balance Scale contribute to the scale’s reliability. The convergent validity of the scale is evidenced by the composite reliability (CR) value being higher than the average variance extracted (AVE) value, and the AVE value being greater than 0.5 [35]. Accordingly, it was determined that the CR and AVE values of the scale are at acceptable levels. These values confirm that the convergent validity of the Artificial Intelligence - Digital Life Balance Scale has been achieved.

### Confirmatory Factor Analysis

The six-factor structure obtained from the Exploratory Factor Analysis (EFA) was examined for model-data fit using Confirmatory Factor Analysis (CFA). In evaluating the CFA results, commonly used fit indices in the literature include χ²/sd, GFI, AGFI, NFI, NNFI, IFI, CFI, RMSEA, RMR, and SRMR [50, 51]. The acceptable cutoff points for these goodness-of-fit indices are as follows: χ²/sd ≤ 3; GFI, AGFI, CFI, NFI, and TLI ≥ 0.90; SRMR, RMSEA ≤ 0.08 [46, 51, 52].

**Fig. 2:**
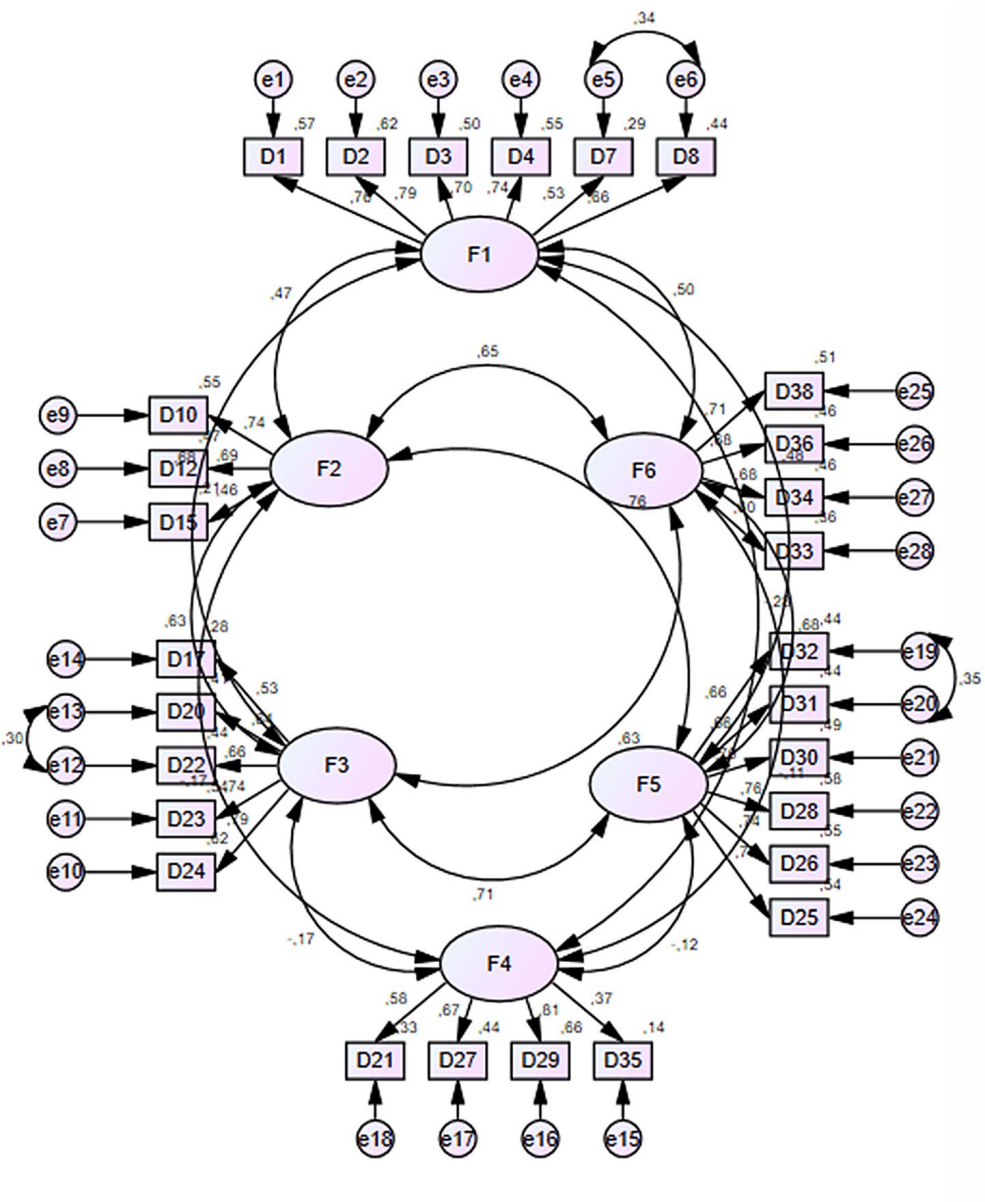
Confirmatory factor analysis final model

The goodness-of-fit indices obtained from the CFA are as follows: χ²/sd = 2.11, GFI = 0.91, AGFI = 0.90, NFI = 0.90, NNFI/TLI = 0.89, IFI = 0.99, CFI = 0.90, RMSEA = 0.06, and SRMR = 0.06. These results indicate that the fit indices of the scale are within acceptable ranges. The CFA results confirm that the six-factor structure is consistent with the theoretical model. The fact that the model fit indices (e.g., RMSEA = 0.06, CFI = 0.90) fall within acceptable limits demonstrates that the scale is psychometrically adequate and reliable. These findings suggest that the scale can be reliably used in both clinical contexts (e.g., screening for technology addiction) and research contexts (e.g., studies on digital life balance). The model obtained from the CFA is presented in Fig. 2 below.

To determine the temporal consistency of the scale, it was administered to 86 students with a 30-day interval. It was found that the correlation coefficients between the first and second administrations of the scale’s factors ranged between 0.86 and 0.91. A positive and highly significant relationship was observed between the pre-test and post-test measurements of the scale. Additionally, Intraclass Correlation Coefficients (ICC) were calculated for the test-retest analysis and are presented in Table 3. Based on these findings, it can be stated that the scale demonstrates temporal consistency.

**Table 3:** Intraclass correlation coefficients of the artificial intelligence - digital life balance scale (ICC)

Item	Intraclass correlation coefficients (ICC)
1	0.90
2	0.91
3	0.90
4	0.88
7	0.90
8	0.89
10	0.92
12	0.93
15	0.89
17	0.93
20	0.94
22	0.90
23	0.93
24	0.96
21	0.93
27	0.90
29	0.89
35	0.95
25	0.93
26	0.90
28	0.90
30	0.94
31	0.93
33	0.92
34	0.90
36	0.91
38	0.92

Before conducting the measurement invariance analyses, the model-data fit values for the original factor structure of the Artificial Intelligence - Digital Life Balance Scale with respect to gender are presented in Table 4.

**Table 4:** Fit indices for subgroups of the artificial intelligence - digital life balance scale

Groups	x²	sd	RMSEA (%90 GA)	SRMR	CFI	TLI
Women	701.572	332	0.060 (0.054–0.066)	0.061	0.90	0.90
Men	696.145	332	0.061 (0.055–0.067)	0.059	0.90	0.90

Note: df = degrees of freedom, confidence intervals (%90) for RMSEA values are provided in parentheses

Upon examining Table 4, it is observed that the fit indices of the measurement model of the Artificial Intelligence - Digital Life Balance Scale for the gender variable fall within the ranges commonly accepted in the literature for evaluating model-data fit. In this context, the six-factor structure of the Artificial Intelligence - Digital Life Balance Scale is found to be consistent with the data obtained from all subgroups. In other words, it provides evidence that the original factor structure is confirmed in each subgroup and that construct validity is achieved in each subgroup.

### Findings on Measurement Invariance

The measurement invariance of the Artificial Intelligence - Digital Life Balance Scale with respect to the gender variable was examined using multi-group confirmatory factor analyses. In this context, configural, metric, and scalar invariance models were tested for the variable. The findings regarding measurement invariance are presented in Table 5.

**Table 5:** Multigroup confirmatory factor analysis results for the artificial intelligence - digital life balance scale

Variable	χ2	sd	RMSEA	CFI	SRMR	Δχ2	Δsd	*p*	ΔCFI	ΔRMSEA	ΔSRMR
Gender											
Configural	272.14	100	0.064	0.909	0.060						
Metric	**279.85**	105	0.063	0.904	0.063	**14.32**	8	0.08	0.003	**0.002**	**0.007**
Scalar	**293.45**	**119**	**0.061**	**0.900**	**0.065**	**13.95**	8	0.13	0.002	**0.002**	**0.002**

Upon examining Table 5, it can be stated that the fit indices used to evaluate model fit for the configural invariance stage are within acceptable limits for all groups (RMSEA < 0.08, CFI > 0.90, NFI > 0.90, NNFI > 0.90, IFI > 0.90). In the configural invariance stage, since the factor loadings, inter-factor correlations, and error variances were freely estimated across subgroups, it can be concluded that the structure of the measurement model of the Artificial Intelligence - Digital Life Balance Scale is similar across gender subgroups. With the configural invariance stage completed, the analysis proceeded to the next stage of metric invariance.

In the metric invariance stage, the constraint of equal factor loadings across subgroups was imposed. The obtained fit indices were evaluated, and it was determined that the model successfully fit the data. To assess metric invariance, the differences in CFI and RMSEA values between the configural and metric invariance stages were analyzed, and it was found that ∆CFI and ∆RMSEA were within acceptable ranges (∆CFI ≤ 0.01; ∆RMSEA ≤ 0.015). This result indicates that the factor loadings of the variables included in the model did not differ across gender subgroups. After establishing metric invariance, the final stage involved testing scalar invariance by constraining factor structures, factor loadings, and item intercepts to be equal across groups. The fit indices for scalar invariance demonstrated that scalar invariance was adequately achieved. The models for scalar and metric invariance were compared, and the obtained values were found to fall within the criterion values proposed by [53]. The data from these model comparisons confirm that the Artificial Intelligence - Digital Life Balance Scale exhibits configural, metric, and scalar measurement invariance across female and male student subgroups.

## Discussion, Conclusion, and Recommendations

This study examines the psychometric properties of the Artificial Intelligence - Digital Life Balance Scale, developed using ChatGPT, providing significant insights into the validity and reliability of artificial intelligence applications. In this context, conducting validity and reliability analyses of the Artificial Intelligence - Digital Life Balance Scale developed by ChatGPT offers valuable contributions to both academic literature and practitioners, paving the way for the development of future psychometric scales. Analyses were performed to establish the validity and reliability of the Artificial Intelligence - Digital Life Balance Scale, created via ChatGPT, to measure university students’ perceived levels of digital life balance. The results of the analyses revealed that the scale has a six-factor structure, with factor loadings of the scale items ranging between 0.53 and 0.81, common factor variances varying between 0.48 and 0.69, and the six-factor structure explaining 60.83% of the total variance. The corrected item-test correlation coefficients of the scale were found to range between 0.54 and 0.85. Compared to the initially designed five-dimensional theoretical model, the six-factor structure of the scale provides a more detailed framework. The factors were named as follows: (1) Frequency and Duration of Digital Device Use, (2) Psychological and Social Impacts, (3) Physical Health Impacts, (4) Academic Barriers, (5) Academic Support, and (6) Technology Dependence. The emergence of the sixth factor (Technology Dependence) indicates that individuals’ levels of dependence on technology should be evaluated independently of the contribution of digital resources to academic performance. This new structure better reflects the complex nature of digital life balance and enables more nuanced analyses in clinical applications (e.g., assessing addiction risk). However, the theoretical basis for this divergence should be explored in greater detail in future studies [36]. The Confirmatory Factor Analysis (CFA) conducted on the six-dimensional scale revealed that the goodness-of-fit indices, when evaluated against the values established in the literature [46, 51, 52], indicate an acceptable fit. It was found that the Cronbach’s alpha coefficients for the scale’s dimensions ranged between 0.68 and 0.87, and the McDonald’s Omega coefficients also ranged between 0.68 and 0.87. The calculated Composite Reliability (CR) and Average Variance Extracted (AVE) values for the scale were determined to be within the acceptable limits established in the literature. Based on the results of the test-retest analysis, it can be concluded that the developed scale is stable over time.

After conducting validity and reliability analyses for the Artificial Intelligence - Digital Life Balance Scale, the measurement invariance of the scale with respect to the gender variable was examined. Within the scope of measurement invariance, configural, metric, and scalar invariance models were sequentially tested for each variable. The obtained values indicate that the Artificial Intelligence - Digital Life Balance Scale exhibits configural, metric, and scalar measurement invariance across gender. This scale provides clinicians with a valuable assessment tool, particularly for evaluating technology addiction and mental health issues. For instance, the “Psychological and Social Impacts” subscale can be effectively used to assess levels of anxiety, stress, or social isolation resulting from technology use. In clinical practice, this scale can serve as a guiding tool for clinicians in cognitive behavioral therapy (CBT) processes, in evaluating individuals at risk of technology addiction, or in measuring the effectiveness of digital detox programs. Specifically, the reverse-scored items of the scale can be utilized as a screening tool to identify the adverse effects of technology use on mental health, enabling clinicians to design tailored interventions [9]. Furthermore, high scores on the scale may indicate a healthy digital life balance, allowing mental health professionals to develop strategies to support individuals in cultivating a lifestyle harmonious with technology use. Given the universal nature of issues related to digital life balance, it can be argued that this scale holds significant potential for international application. Although the current study focused on cultural characteristics such as widespread smartphone use (over 95%; [5]) and prevalent social media engagement among university students in Türkiye, examining the scale’s validity across different cultural and demographic groups is of great importance. For instance, validity and reliability analyses of the scale could be conducted in diverse populations, such as adolescents, working adults, or elderly individuals in European or Asian countries. Comparative studies, for example, with the Internet Addiction Test developed in the United States [54] or social media addiction scales used in China [12], could highlight the advantages of this scale’s multidimensional structure. Testing the scale across different socioeconomic levels and cultural contexts could further its development to contribute to global mental health research. Such studies could not only facilitate the scale’s recognition as an assessment tool for addressing digital addiction on a global scale but also support the development of intervention strategies. The ethical and methodological dimensions of using AI models like ChatGPT in the scale development process enhance the innovative aspect of the study while also sparking certain debates. From an ethical perspective, the most critical issue is the risk that these models may learn from biased datasets, which could compromise the impartiality of scale items [32]. For instance, if the system’s training data contains biases toward specific cultural or demographic groups, the generated items may reflect these biases. To mitigate this risk in the present study, the scale items generated by AI were subjected to expert evaluation and rigorously reviewed for cultural appropriateness. Additionally, the originality of AI-generated content may be limited by the reconfiguration of training data, which raises questions about the theoretical originality of the scale [55]. From a methodological standpoint, while AI’s rapid item generation and accessibility democratize the scale development process, the lack of critical thinking underscores the crucial importance of human expert oversight. In this study, the expert evaluation process enhanced the theoretical consistency and psychometric robustness of the scale. For the future, it is recommended that ethical guidelines for AI-assisted scale development processes (e.g., data transparency, bias monitoring) be defined within a clearer framework.

The findings of this study demonstrate that, compared to other scales in the field of digital addiction, the multidimensional structure of the developed scale offers significant advantages. For instance, while commonly used scales such as the Smartphone Addiction Scale [22] and the Internet Addiction Test [54] typically focus on addictive behaviors, the scale developed in this study adopts a holistic approach, addressing the psychological, social, physical, and academic dimensions of digital life balance. The multidimensional structure of the scale provides a more comprehensive and detailed assessment compared to the narrow scope of most existing scales, which primarily focus on measuring pathological usage. Another innovative aspect of this study is the use of artificial intelligence technologies in the scale development process. This approach facilitated the rapid and accessible production of the scale, presenting an innovative alternative to traditional scale development processes. The practical applications of the scale offer a wide range of uses for mental health professionals, educators, and policymakers. Clinical psychologists and psychiatrists can effectively utilize the scale to identify individuals at risk of technology addiction and to develop tailored intervention plans. For educators and school counselors, the scale serves as a useful tool for assessing students’ digital habits and evaluating the effectiveness of school-based digital wellness programs. Policymakers can leverage the scale’s results as a data source to develop public policies promoting digital well-being (e.g., digital awareness campaigns). For future research, it is recommended that the scale be tested across different age groups (e.g., adolescents or older adults) and occupational groups (e.g., office workers, digital content creators). Additionally, longitudinal studies examining changes in digital life balance over time could provide valuable insights into the dynamic nature of the scale. For example, the effects of increased digitalization trends post-pandemic [56] could be investigated using this scale.

The learning capacity of ChatGPT demonstrates its potential to develop meaningful and reliable scales without human intervention. The findings obtained for the Digital Life Balance Scale designed by ChatGPT in this study indicate that a valid and reliable tool has been developed to measure the digital life balance of university students. The research is expected to serve as a valuable resource for studies examining the digital life balance levels of university students in Türkiye. Furthermore, the validity and reliability analyses of this scale, developed with the contribution of ChatGPT, can offer significant contributions to both academic literature and applied studies, potentially guiding future psychometric scale development processes.

ChatGPT, a conversational AI application developed and supervised by OpenAI, operates with a large language model optimized through reinforcement learning methods, and its generative potential is anticipated to contribute to scale development processes [57, 58]. However, the fact that AI operates based on existing data may pose a limitation in terms of content originality. AI models developed on deep learning algorithms are trained on large datasets, enabling them to learn patterns and relationships within the data. However, this means that the content generated by AI may not be entirely original but rather a reconfiguration or reformulation of the information in the training data. In this context, the generated content may often be a derivative of existing data. Additionally, the lack of critical thinking in AI is considered a significant limitation [55, 59].Derin öğrenme algoritmalarına dayalı yapay zekâ modellerinin büyük veri setleriyle eğitilmesi, bu sistemlerin bilgi işleme ve örüntü tanıma becerilerini ileri düzeye taşımaktadır. Ancak bu güçlü öğrenme süreci, beraberinde bazı tartışmaları da getirmektedir. Özellikle yapay zekâ, eğitim verilerinde yer alan bilgileri yeni biçimlerde sunabilir; yeniden ifade edebilir ya da çeşitli şekillerde birleştirerek kullanabilir. Bu da, üretilen içeriklerin çoğu zaman tamamen özgün olmaktan çok, mevcut verilerin farklı bir türevi niteliğinde olmasına yol açabilir. Bu bağlamda, yaratıcı üretimin önem kazandığı alanlarda, yapay zekânın sağlayabileceği katkılar kadar, taşıdığı sınırlamalar da dikkatle değerlendirilmelidir.

Furthermore, the limited critical thinking capacity of artificial intelligence stands out as a significant disadvantage. Humans consider diverse perspectives when approaching information, analyze conflicting data to synthesize insights, and develop original ideas. In contrast, AI models typically generate outputs based on historical data and may fall short in evaluating abstract, complex, or ambiguous situations. This limitation highlights that AI cannot fully replace human expertise, particularly in problems requiring creative thinking or complex decision-making processes [55, 59, 60]. Therefore, AI-supported applications, especially in sensitive processes such as scale development, must be used with caution and under controlled conditions.

The limitations of the study should be considered when interpreting the results. First, the sample, consisting solely of students from a university in eastern Türkiye, limits the scale’s generalizability to the broader population. The homogeneity of the sample may not reflect the perceptions of digital life balance among individuals from different age groups, socioeconomic levels, or occupational groups. Second, while the scale addresses specific aspects of digital life balance, such as device usage, psychological impacts, physical health, academic performance, and addiction, it does not cover other significant factors, such as work-life balance or concerns about digital privacy. This can be considered a factor limiting the scale’s scope. Finally, the limitations of ChatGPT in the scale development process, particularly the risk of generating items lacking theoretical grounding or full alignment with cultural contexts, constitute the methodological boundaries of the study. For instance, without expert evaluation, the items generated by AI could have been theoretically deficient or contextually inappropriate [32]. These limitations indicate the need to test the scale across diverse demographic groups and cultural contexts and to anchor it within a broader theoretical framework.

## Data Availability

The data used to support the findings of this study are available upon request from the corresponding author or can be accessed through the Harvard Dataverse link.

## Declarations

### Statement on the Use of Artificial Intelligence

The authors state that no specific or personally identifiable data were provided to artificial intelligence during the preparation of this article. It is acknowledged that transferring personal data to AI systems for research, authorship, or article publication purposes could raise ethical concerns regarding privacy and confidentiality. In the use of AI, risks arising from biased datasets and limitations in the originality of generated content were taken into account. For instance, as potential biases in ChatGPT-4’s training data could affect the impartiality of scale items, the items were subjected to expert review to assess their ethical appropriateness [32]. Additionally, the lack of transparency in the data processing procedures of AI models is an ethical point of discussion, and this study adhered strictly to data privacy principles. In ChatGPT-4’s data processing, risks such as data leakage or misuse were carefully addressed. In this context, only general and theoretical prompts were used in accordance with OpenAI’s privacy policies, and no sensitive or personal data were shared. To ensure the ethical appropriateness of AI-generated content, the items were reviewed by academic experts and scrutinized for cultural or societal biases [61]. All data used were processed in compliance with relevant privacy and ethical guidelines.
